# RETRACTED: Hassanin et al. Design and Synthesis of New Hydantoin Acetanilide Derivatives as Anti-NSCLC Targeting EGFR^L858R/T790M^ Mutations. *Pharmaceuticals* 2022, *15*, 857

**DOI:** 10.3390/ph19111788

**Published:** 2026-11-11

**Authors:** Moamen A. Hassanin, Muhamad Mustafa, Mohammed A. S. Abourehab, Heba A. Hassan, Omar M. Aly, Eman A. M. Beshr

**Affiliations:** 1 Department of Medicinal Chemistry, Faculty of Pharmacy, Minia University, Minia 61519, Egypt; moamenawad15@gmail.com (M.A.H.); hebahassan2009@live.com (H.A.H.);; 2 Department of Medicinal Chemistry, Faculty of Pharmacy, Deraya University, Minia 61111, Egypt; 3 Department of Pharmaceutics, Faculty of Pharmacy, Umm Al-Qura University, Makkah 21955, Saudi Arabia; maabourehab@uqu.edu.sa; 4 Department of Medicinal Chemistry, Faculty of Pharmacy, Port Said University, Port Said 42511, Egypt

The journal retracts the article titled “Design and Synthesis of New Hydantoin Acetanilide Derivatives as Anti-NSCLC Targeting EGFR^L858R/T790M^ Mutations” [1], cited above.

Following publication, concerns were brought to the attention of the Editorial Office regarding the integrity of the figures presented in the article [1].

In accordance with the journal’s standard procedures, the Editorial Office and Editorial Board conducted an investigation, which confirmed indications of inappropriate editing and panel duplication between Figure 7D of this publication, Figure 4A published in [2], and Figure 6A published in [3], by different authorship groups, reporting different experimental conditions. Although the authors cooperated with the investigation, the Editorial Board determined that the explanations and supporting documentation provided were insufficient to resolve the concerns identified, and the required changes were deemed too extensive to be addressed through a correction. Consequently, the Editorial Board lost confidence in the reliability of the findings and decided to retract this publication [1], in accordance with MDPI’s retraction policy.

This retraction was approved by the Editor-in-Chief of the *Pharmaceuticals* journal.

The authors have been informed of this retraction and have indicated their disagreement with this decision.
